# Mechanism Investigation of the Improvement of Chang Run Tong on the Colonic Remodeling in Streptozotocin-Induced Diabetic Rats

**DOI:** 10.1155/2016/1826281

**Published:** 2015-12-28

**Authors:** Hong Sha, Dong Zhao, Xiaolin Tong, Hans Gregersen, Jingbo Zhao

**Affiliations:** ^1^China-Japan Hospital, Beijing 100029, China; ^2^Guang'anmen Hospital, China Academy of Chinese Medical Sciences, Beijing 100053, China; ^3^Bioengineering College of Chongqing University, Chongqing 400044, China; ^4^Department of Clinical Medicine, Aarhus University, 8200 Aarhus N, Denmark

## Abstract

Previous study demonstrated that Chang Run Tong (CRT) could partly restore the colon remodeling in streptozotocin- (STZ-) induced diabetic rats. Here we investigated the mechanisms of such effects of CRT. Diabetes was induced by a single injection of 40 mg/kg of STZ. CRT was poured into the stomach by gastric lavage once daily for 60 days. The remodeling parameters were obtained from diabetic (DM), CRT treated diabetic (T1, 50 g/kg; T2, 25 g/kg), and normal (Con) rats. Expressions of advanced glycation end product (AGE), AGE receptor, transforming growth factor-*β*1 (TGF-*β*1), and TGF-*β*1 receptor in the colon wall were immunochemically detected and quantitatively analyzed. The association between the expressions of those proteins and the remodeling parameters was analyzed. The expressions of those proteins were significantly higher in different colon layers in the DM group (*P* < 0.05, *P* < 0.01) and highly correlated to the remodeling parameters. Furthermore, the expressions of those proteins were significantly decreased in the T1 group (*P* < 0.05, *P* < 0.01) but not in the T2 group (*P* > 0.05). The corrective effect on the expressions of those proteins is likely to be one molecular pathway for the improvement of CRT on the diabetes-induced colon remodeling.

## 1. Introduction

Constipation is very common in the general population [1] (Belsey et al., 2010) and affects the quality of human life [2]. Colonic and rectal sensory-motor function and biomechanical properties are now strongly implicated in the pathogenesis of constipation [3, 4]. However, the pathophysiological mechanisms underlying chronic constipation remain to be explored [5, 6]. Furthermore constipation is difficult to treat and has a high recurrence rate [7, 8].

The traditional Chinese medicine has good clinical effects on constipation [9]. During the past several years, the authors have through clinical observation found that the Chang Run Tong (CRT) produced by China-Japan Friendship Hospital could effectively treat senile constipation. From Chinese medicine point of view, CRT regulates qi, relieves stagnation, and lubricates the bowel. However, from the Western medicine point of view, the mechanism of CRT in the treatment of constipation is unclear.

The gastrointestinal (GI) tract is functionally subjected to dimensional changes. Hence, biomechanical properties such as the stress-strain relationship are of particular importance [10]. The remodeling of the mechanical properties reflects the changes in the tissue structure that determine a specific motor dysfunction. A previous study has demonstrated that experimental diabetes could induce colon morphological and biomechanical remodeling [11]. Following the development of diabetes, the colonic wall became thicker and the stiffness of the wall increased in a time-dependent manner. Such remodeling plays an important role in diabetic GI complications, including constipation [12]. Therefore, the streptozotocin-induced diabetic rat model is a good model to study the effect of drugs in the treatment of constipation on the morphological and biomechanical remodeling of the GI tract caused by diseases. Using this model, we recently demonstrated that CRT could partly restore the morphometric and biomechanical remodeling of colon in streptozotocin- (STZ-) induced diabetic rats [13]. However the molecular pathways of CRT effect are unclear.

Advanced glycation end products (AGEs) are formed physiologically, increased with aging, and accelerated in diabetes [14]. AGEs can lead to structural and functional changes by direct target protein or through their receptor (RAGE) [15]. AGEs and RAGE have been demonstrated to play an important role in diabetic complications including the complications of the gastrointestinal tract [16]. TGF-*β*1 has been considered to be core factor in the occurrence and development of diabetic nephropathy (DN) [17, 18]. In recent years, the relationship between TGF-*β*1 and other diabetic complications has been gradually noticed [19, 20]. But there is lack of study about the relationship between TGF-*β*1 and TGF-*β*1 receptor with the diabetic colonic histomorphological and biomechanical remodeling. Some studies have demonstrated that the association existed in the expressions of AGE/RAGE and TGF-*β* during the development of diabetes [21, 22].

In order to investigate the mechanism of CRT in the improvement on the morphometric and biomechanical remodeling of the colon in streptozotocin- (STZ-) induced diabetic rats, the expressions of advanced glycation end product (AGE), AGE receptor (RAGE), transforming growth factor-*β*1 (TGF-*β*1), and TGF-*β* receptor were detected in the colon wall. The association between those proteins expression and the histomorphometric and biomechanical parameters was analyzed as well.

## 2. Materials and Methods

### 2.1. Animal Model and Groups

Forty male Sprague-Dawley rats weighing 220–250 g were included in this study. Thirty rats were made diabetic by a single tail-vein injection of 40 mg/kg STZ (Sigma-Aldrich, China). This dose of STZ resulted in a random blood glucose level (≥16.7 mmol/L) in 90% of the rats 7 d after injection. The remaining 10% of rats were excluded from this study. Twenty-seven STZ-induced diabetic rats were subdivided into three groups (*n* = 9 in each group), namely, the diabetic control group (DM), the high-dose CRT group (T1), and the low-dose CRT group (T2). Another ten rats of similar age and body weight from the same vendor were used as the nondiabetic control group (Con).

### 2.2. Drugs and Administration Methods

CRT is composed of* Radix Angelicae Sinensis, Radix Cyathulae, Herba Cistanches, Rhizoma Alismatis, Rhizoma Cimicifugae, Fructus Aurantii Immaturus, Rhizoma Atractylodis Macrocephalae, Semen Arecae Prepareta*, and hemp seed provided by China-Japan Hospital, The Ministry of Health of the People's Republic of China. The medicine was directly injected into the stomach lumen by gastric gavage once daily from the beginning of the experiment. The dosage was 50 g/kg/day for T1 and 25 g/kg/day for T2. The rats of the DM and Con groups were only given physiological saline.

### 2.3. Experimental Procedures and Sampling

The body weight and blood glucose levels were measured at 2-week intervals after the start of the experiment. The experimental period was 60 d. At the ending of the experiment, the rats fasted overnight and then were anesthetized with 4% chloral hydrate (10 mL/kg, ip). Following laparotomy, the whole colon was harvested. After the lumen of the segments was gently cleaned with saline, the length and the wet weight were measured. The colonic segment was divided into three parts: A 2 cm long tissue was cut from proximal end of the segments and fixed in 10% formalin for immunohistochemistry examination. Then a 1 cm long part was cut and used for the zero-stress state experiment and the remaining part was used for the distension test. The results of zero-stress state and the distension test have been reported in our previous paper [13]. The parameters of morphometric properties, residual strains, and stress-strain of the wall in colonic segments were adopted from our previous paper [13] and used for correlation analysis with the expressions of different proteins used in the present paper.

### 2.4. Immunohistochemistry Staining

#### 2.4.1. Tissue Pretreatment

The tissue samples for immunohistochemistry were fixed in 10% phosphate-buffered formalin for 24 h and embedded in paraffin. Five-micron sections were cut perpendicular to the mucosa surface and placed in a water bath at 40°C. Thereafter, sections were transferred onto pretreated microscopic slides which electrostatically attracted formalin fixed tissue and bound them to the slides. After drying the slides completely at room temperature, they were treated in an oven at 37°C overnight to enhance the attachment of tissue to the slides. The sections were deparaffinized two times in xylene, 15 min per time, and rehydrated in 100%, 95%, 90%, 80%, 70%, 60%, and 50% ethanol two times, respectively, 3 sec per time, followed by rinsing for 10 min and washing in 0.01 M PBS (pH 7.4).

#### 2.4.2. AGE

After treatment with H_2_O_2_ (3% in ethanol, room temperature, 15 min) and proteinase K (100 *μ*g/mL, 100 *μ*L, 37°C, 20 min), the sections were incubated with 5% BSA-PBS buffer (room temperature, 30 min) for blocking nonspecific staining. Afterwards, the sections were incubated with the primary anti-AGE antibody (Abcam, 1 : 100, diluted in 1% BSA-PBS) or normal mouse IgG (250 *μ*g/mL) pretreated with excessive CML (1 : 250, diluted in 1% BSA-PBS, negative control) over night at 4°C. The sections were then washed and incubated with LINK (biotinylated anti-rabbit and anti-mouse immunoglobulin) and streptavidin peroxidase (streptavidin conjugated with horseradish peroxidase), respectively, at room temperature for 10 min (both are part of reagents of LSAB2 System-HRP, products of Dako Company, Denmark). Then the peroxidase activity was visualized by incubating the sections in substrate working solution containing hydrogen peroxide and 3,3′-diaminobenzidine tetrahydrochloride at room temperature for 5 min. The sections were rinsed for 10 min, counterstained with Mayer hematoxylin for 1 min, treated in HCl-ethanol for 3 sec, and dehydrated in 80%, 90%, 95%, and 100% ethanol for 3 sec, respectively. Then the slides were immersed in xylene for 15 min two times and mounted.

#### 2.4.3. RAGE

The primary anti-RAGE antibody was produced in rabbits immunized with a synthetic peptide corresponding to a sequence at the N-terminal of human RAGE (Sigma). Only two amino acids are different from the related rat sequence. Instead of treating sections with proteinase K for RAGE immunostaining, the sections were boiled in 10 mM citrate buffer (pH 6.0) for 18 min for retrieving antigen. Normal rat lung was used as positive control since RAGE is highly expressed in the lung [23]. The primary antibody was diluted (1 : 80) with 1% BSA-PBS and normal rabbit serum (diluted 1 : 60) pretreated with excessive soluble RAGE was used as negative control. Other processes were similar to the AGE immunostaining.

#### 2.4.4. TGF-*β*1 and TGF-*β*1 Receptor

The primary TGF-*β*1 antibody (BA0290) and TGF-*β*1 receptor antibody (BA0526-2) were obtained from Wuhan Boster Biological Engineering Co., Ltd. They were all diluted (1 : 100) with 1% BSA-PBS. The second antibody is HRP-goat anti-rabbit IgG and was diluted (1 : 150) with 1% BSA-PBS. The sections were placed in 3% H_2_O_2_ (AR1108) at room temperature for 5–10 minutes to inactivate endogenous enzymes. The sections were rinsed with distilled water for 3 times. Hot fix antigen: The sections were immersed in 0.01 M citrate buffer (AR0024, pH 6.0) or 0.02 M PBS (AR0030, pH 7.2–7.6) and heated to boiling using electricity or microwave for retrieving antigen. This process was repeated one or two times and the interval is 5–10 minutes. Then the slides were naturally cooled to room temperature. The sections were incubated with 5% BSA blocking solution (AR0004) (37°C, 30 minutes) for blocking nonspecific staining. Then the excess liquid was shanked off from the slides (do not wash) and incubated with diluted primary antibody over night at 4°C or 2 hours at 37°C. The slides were rinsed with PBS (pH 7.2–7.6) for 3 times (each time lasts 5 minutes). Then the slides were incubated with corresponding second antibodies (37°C, 30 minutes). The slides were rinsed again for 3 times (each time lasts 5 minutes). The slides were incubated with SABC (37°C, 30 minutes) and washed for 4 times (each time lasts 5 minutes) with PBS (pH 7.2–7.6). Then the peroxidase activity was visualized by incubating the sections with DAB visualized kit (AR1022, taking 1 drop from each of A, B, and C reagent and mixing into 1 mL of distilled water) for about 15 minutes at room temperature. The slides were washed with distilled water and counterstained with hematoxylin (AR0005). Then the slides were dehydrated, transparent, and mounted.

#### 2.4.5. Image Analysis

AGE, RAGE, TGF-*β*1, and TGF-*β*1 receptor are shown brown staining, but such color does not appear in the negative control slides, indicating that the staining is specific. To minimize errors, 6 to 10 photographs from different locations of the same layer in each slide were randomly taken. After that, different parts were saved as individual image files. The region of interest (ROI) was defined using Sigmascan Pro 4.0 image analysis software. The color due to 3,3′-diaminobenzidine staining was distinguished in the ROI using intensity thresholds. Finally the images were exported as binary images. The total area and area fraction of AGE, RAGE, TGF-*β*1, and TGF-*β*1 receptor positive staining were calculated by a Matlab program (Matlab 6.5, The MathWorks Inc., USA). Then the fraction of AGE, RAGE, TGF-*β*1, and TGF-*β*1 receptor in mucosa, muscle, and submucosa layers were computed as fraction of protein expressions = immunopositive area/total measured area.

### 2.5. Statistical Analysis

The data were representative of a normal distribution and accordingly the results were expressed as means ± SEM. Student's *t*-test and analysis of variance (ANOVA) were used to detect differences between parameters and groups (Sigmastat 2.0). Linear regression analysis was used to demonstrate possible association between the AGE, RAGE, TGF-*β*1, and TGF-*β*1 receptor expressions and histomorphometric and biomechanical parameters. The results were regarded as significant when *P* < 0.05.

## 3. Results

### 3.1. General Data and Morphometry Data

The blood glucose, body weight, the wet weight per unit length, no-load wall thickness, and cross section wall area measured at the end of the experiment are shown in Table 1. The blood glucose level was about 4-fold higher in the DM group compared with that of the Con group (*P* < 0.01). The body weight in the DM group was nearly 50% lower than that in the Con Group. Compared with the DM group, the blood glucose level in the T1 group (*P* < 0.05) was lower but that in the T2 group (*P* > 0.05) was higher. The body weight did not differ among the DM, T1, and T2 groups (*P* > 0.05). The wet weight per unit length, no-load wall thickness, and cross section wall area of the colonic segments were significantly higher in the diabetic group compared with those of the Con group (*P* < 0.01). After treatment with T1, these parameters significantly decreased in the two segments (*P* < 0.05 and *P* < 0.01); however, they did not significantly change in the T2 group (*P* > 0.05) with the exception of the wall thickness (*P* < 0.05).

### 3.2. Biomechanical Data

The biomechanical parameters of the colon segment obtained from previous study [13] were shown in Table 2. The opening angles were significantly higher in the DM group compared with those in the Con group (*P* < 0.01). Treatment with a high dosage of CRT decreased the opening angle significantly (*P* < 0.05); the opening angle did not change in the T2 group (*P* > 0.05). A similar trend was found for the inner and outer residual strains; that is, the absolute values of the residual strain were significantly higher in the DM group compared with those in the Con group (*P* < 0.05, *P* < 0.01). Treatment with a high dosage of CRT (T1 group) partially reversed the changes of residual strain (*P* < 0.05). Computation of constant *α* showed a significant difference between the DM group and the Con group (*P* < 0.05). High-dosage CRT (T1) treatment significantly decreased the stiffness of the colonic wall in both circumferential (*P* < 0.05) and longitudinal (*P* < 0.05) directions. Low-dosage CRT treatment (T2) did not show improvement in the stiffening of the colonic wall caused by diabetes (*P* > 0.05).

### 3.3. Fractions of AGE, RAGE, TGF-*β*1, and TGF-*β*1 Receptor Expressions in Different Groups

**Figure 1:**
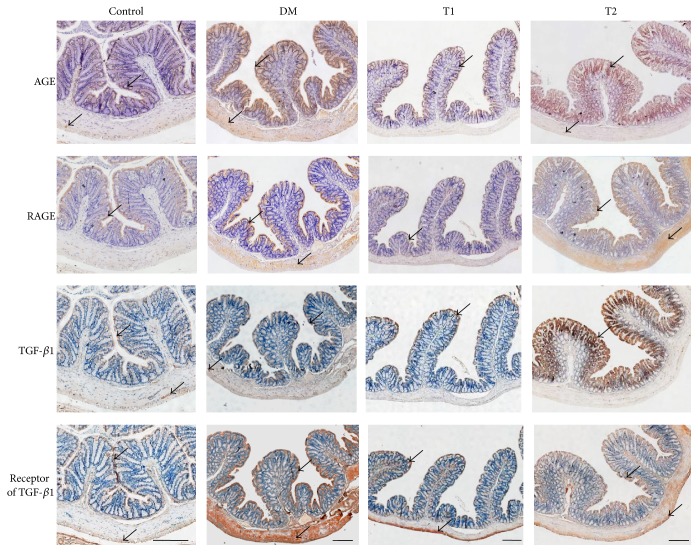
The representative samples of immunohistochemical staining for AGE, RAGE, TGF-*β*1, and TGF-*β*1 receptor in different groups. The brown color indicated positive staining and the arrows were to point the examples where the immunostaining is positive. The staining of all proteins was stronger in the muscle layer than other layers. The staining of AGE, RAGE, TGF-*β*1, and TGF-*β*1 receptor in different layers was stronger in the DM group than in Con group. Treatment with a high dosage of CRT (T1 group) partially reversed the abnormal expressions of different proteins in the different layers of the colon wall; however they did not significantly change in the T2 group. Bar = 1 mm.

The fraction of AGE, RAGE, TGF-*β*1, and TGF-*β*1 receptor expressions in the different layers of the colon in different groups were shown in Table 3. The representative samples of immunohistochemical staining were shown in Figure 1. Generally the expression of all proteins was stronger in the muscle layer than other layers. The expressions of AGE, RAGE, TGF-*β*1, and TGF-*β*1 receptor in different layers were upregulated in the DM group than in Con group. Treatment with a high dosage of CRT (T1 group) partially reversed the abnormal expressions of different proteins in the different layers of the colon wall; however they did not significantly change in the T2 group. The detail results including *P* values were shown in Table 3.

### 3.4. Correlation Analysis Results

#### 3.4.1. Single Linear Correlation Analysis

In order to analyze the expressions of AGE, RAGE, TGF-*β*1, and TGF-*β*1 receptor with other parameters, the linear regression analysis was separately done on the expressions of AGE, RAGE, TGF-*β*1, and TGF-*β*1 receptor in different layers of the colon wall with blood glucose level, body weight, wet weight per unit length of the colon, wall thickness, wall cross-sectional area, opening angle, inner residual strain, outer residual strain, circumferential material constant *α*, and longitudinal material constant *α*. It was shown that expressions of AGE, RAGE, and TGF-*β*1 receptor correlated with most of the other parameters.  Table 4 listed some results of the correlation. AGE and RAGE in different layers were highly correlated with glucose level, wall thickness, wall area, opening angle, and outer residual strain. AGE and RAGE in muscle layer correlated with circumferential material constant *α*, whereas RAGE in muscle and submucosa layers correlated with longitudinal material constant *α*. TGF-*β*1 receptor in different layers correlated with most parameters, whereas it was only found that the TGF-*β*1 in muscle layer correlated with glucose level, body weight, and circumferential and longitudinal material constant *α*. The details of correlation equations and values of *R* and *P* were shown in Table 4.

#### 3.4.2. Multiple Linear Correlation Analysis

In order to determine the main contributor of AGE, RAGE, TGF-*β*1, and TGF-*β*1 receptor expressions in different layers in association with other parameters, and to see interrelation among AGE, RAGE, TGF-*β*1, and TGF-*β*1 receptor expressions, the multiple linear correlation analysis was done.

Table 5 showed the relation between some parameters with the expressions of AGE, RAGE, TGF-*β*1, and TGF-*β*1 receptor in different layers. Glucose level and wall area mainly correlated with AGE and RAGE expressions. Outer residual strain correlated with all proteins expressions in the mucosa layer and with RAGE expressions in the muscle and submucosa layers. Opening angle was mainly determined by the RAGE in mucosa layer, AGE in muscle layer, and TGF-*β*1 receptor in muscle and submucosa layers. Circumferential material constant *α* seems more related to TGF-*β*1 in mucosa and AGE in muscle and submucosa layers, whereas longitudinal constant *α* seems more related to AGE in mucosa, TGF-*β*1 in muscle, and RAGE in submucosa layers. The details of correlation equations and values of *R*, *F*, and *P* were shown in Table 5.

Interrelation among AGE, RAGE, TGF-*β*1, and TGF-*β*1 receptor expressions in different layers was shown in Table 6. AGE and TGF-*β*1 in different layers mostly correlated with their receptors: RAGE and TGF-*β*1 receptor. Similarly RAGE and TGF-*β*1 receptor in different layers also correlated with AGE and TGF-*β*1. However it is interesting to notice that RAGE and TGF-*β*1 receptor in muscle layers were strongly correlated with each other. The details of correlation equations and values of *R*, *F*, and *P* were shown in Table 6.

## 4. Discussion

Previously we demonstrated that CRT could restore the morphometric and biomechanical remodeling of colon in streptozotocin- (STZ-) induced diabetic rats [13]. The main finding found at the present study was that the expression of AGE, RAGE, TGF-*β*1, and TGF-*β*1 receptor was significantly higher in different colon layers in the DM group than in Con group. The expressions of those proteins were highly correlated to the histomorphometric and biomechanical remodeling parameters. Furthermore, the expressions of AGE, RAGE, TGF-*β*, and TGF-*β* receptor were significantly decreased in the T1 group but not in the T2 group.

### 4.1. Diabetes Upregulates Expressions of AGE, RAGE, TGF-*β*1, and TGF-*β*1 Receptor in Rat Colon Wall

Diabetic GI complications are common in long-standing diabetes [24]. Poor control of diabetes can affect any segment of the gut including the colon [25]. Although many studies have demonstrated that diabetic GI complications involved multifactors [26], the mechanisms are not well understood. At present study we demonstrated that the expressions of AGE, RAGE, TGF-*β*1, and TGF-*β*1 receptor were upregulated in the different layers of diabetic colon wall. It indicated that the abnormal expressions of these proteins are related to diabetic GI complications.

AGEs and RAGE accumulated during the development of DM are associated with cardiovascular complication [27], retinopathy [28], and nephropathy [29] as well as GI complications [30]. Recently Chen and coworkers [31, 32] have found that AGE and RAGE are upregulated in the diabetic small intestine and colon for both type 1 and type 2 diabetic animal models. The present study confirms these findings. The accumulated AGEs may affect the tissue structural changes and neuromuscular function of diabetic GI tract through receptor-dependent and receptor-independent pathways [33, 34]. The former modulates cellular functions through ligation of specific cell surface receptors such as RAGE. The latter alters the extracellular matrix architecture by nonenzymatic glycation and the formation of protein cross-links.

Transforming growth factor- (TGF-) *β*1 is a ubiquitously expressed cytokine belonging to a large superfamily of activins/bone morphogenetic proteins [35]. This mediator plays an active role in the processes of wound healing [36] and synthesis of ECM molecules [37]. It was reported that plasma levels of TGF-*β*1 were elevated in NIDDM patients and might contribute to the occurrence of diabetic complications [38]. Indeed, many studies have demonstrated that TGF-*β* strongly contributes to diabetic nephropathy [17, 18], diabetic retinopathy [39, 40], and diabetic neuropathy [41]. In the present study, we first demonstrated that the TGF-*β*1 and TGF-*β*1 receptor were upregulated in the diabetic colon wall. Although no detail molecular pathway was demonstrated in the present study, the increasing TGF-*β*1 may through ligand binding activate TGF-*β*1 receptors and then activate Smad proteins through phosphorylation according to a review article about the role of TGF-*β* in gastrointestinal pathophysiology and modulation of ulcer healing [42].

Analysis for interrelation among AGE, RAGE, TGF-*β*1, and TGF-*β*1 receptor showed that AGE and TGF-*β*1 in different layers mostly correlated with their receptors: RAGE and TGF-*β*1 receptor. It seems easy to understand that the effects of AGE and TGF-*β*1 are through their corresponding receptors. However it is interesting to notice that RAGE and TGF-*β*1 receptor in muscle layers were strongly correlated with each other. There are some studies to investigate the complicated interaction between AGE and TGF-*β*1 with their receptors in the pathological progression of diabetic nephropathy [43, 44] and interstitial fibrosis induced by imbalances in extracellular matrix homeostasis [45]. However interpretation for the relation between RAGE and TGF-*β*1 receptor in the diabetic GI complication is needed to explore.

### 4.2. Diabetes-Induced Morphological and Biomechanical Remodeling of Rat Colon Associates with the Abnormal Expressions of AGE, RAGE, TGF-*β*1, and TGF-*β*1 Receptor

We have previously demonstrated that the histomorphological and biomechanical remodeling occurred in the diabetic rat model [11]; that is, diabetes generated pronounced increases in the colon wall thickness, wall cross-sectional area, the thickness of all layers, the opening angle, the absolute values of residual strain, and the circumferential and longitudinal stiffness of the colon wall. However, the mechanism of such remodeling is not so clear. It was shown from the study of relation between AGEs and vascular wall stiffness that glycation-induced intermolecular cross-links contributed to diabetic vascular stiffening [46]. Evidences showed that AGE formation in arteries was associated with arterial stiffening during aging [47]. Also study found that high serum AGE concentrations are associated with increased arterial stiffness and thickness in patients with diabetes [48]. More recently one study demonstrated that chronic CML ingestion induced arterial stiffness and aging in a RAGE-dependent manner in the mice [49]. The abovementioned studies demonstrated that the morphological and biomechanical remodeling of arteries in diabetes is closely associated with AGE and RAGE. We, in a previous study [16], demonstrated that the most histomorphometric and biomechanical parameters of intestinal wall in the GK diabetic rats were associated with the expression of AGE and RAGE in villi, especially that the highest association was found between the mechanical constant *α* and villous AGE. At the same time, it was also found that the mechanical constant *α* was associated with AGE and RAGE expressions in the crypt. In the present study, we further confirmed that diabetes-induced morphological and biomechanical remodeling of rat colon were also closely associated with the abnormal expressions of AGE and RAGE in the different layers of colon wall.

Data is lacking in relation to the association between TGF-*β*1 and TGF-*β*1 receptor and gastrointestinal morphological and biomechanical remodeling in diabetes. Fleenor and coworkers [50] reported that arterial stiffening with ageing is associated with TGF-*β*1-related changes in adventitial collagen. Few studies demonstrated that TGF-*β*1 increased F-actin levels in single chondrocytes leading to stiffening of cells [51, 52]. In the present study, we first demonstrated that TGF-*β*1 in muscle layer correlated with circumferential and longitudinal material constant *α*, whereas TGF-*β*1 receptor in different layers correlated with most morphological and biomechanical parameters. It is also interesting to notice that RAGE and TGF-*β*1 receptor in muscle layers were strongly correlated with each other. This may indicate that either TGF-*β*1 is an independent contributing factor or TGF-*β*1 and AGE are cocontributors for the morphological and biomechanical remodeling of colon in diabetes. The detail molecular pathways for the effect of AGE and TGF-*β*1 on colonic remodeling in diabetes are needed to further explore.

### 4.3. Corrective Effect of CRT (High Dose) on Those Proteins Expressions Is One of the Molecular Pathways for the Improvement of CRT on the Colon Remodeling in Diabetic Rats

Clinically we found that CRT decoction was very effective to treat senile constipation. Studies have shown that* Herba Cistanches* had laxative activity in the intestine and promoted bowel movement [53].* Semen Arecae Prepareta* promoted gastrointestinal movement, excited cholinergic M receptor, and promoted spontaneous contraction of isolated ileum [54].* Fructus Aurantii Immaturus* enhanced gastrointestinal smooth muscle contraction and intestinal propulsive function [55].* Fructus Cannabis* could shorten the defecation time and significantly increase frequency of bowel movements [56, 57].* Radix Angelicae Sinensis* could promote bowel movement [58]. Therefore, the CRT could regulate GI motility and improve the symptoms in patients with constipation. Previous study has demonstrated that the experimental diabetes could induce colon morphological and biomechanical remodeling [11]. Such remodeling plays an important role in diabetic GI complications including the constipation [12]. In order to investigate the mechanism of CRT to treat the constipation whether or not through the improvement of the morphometric and biomechanical remodeling, we selected STZ-induced diabetic rat model to test the CRT on the morphometric and colonic remodeling induced by diabetes. We have demonstrated that high-dose CRT could partly reverse the diabetes-induced morphological and biomechanical remodeling of colon [13] (Tables 1 and 2). However the mechanism of the CRT effect is not clear. In the present study we demonstrated that the expressions of AGE, RAGE, TGF-*β*1, and TGF-*β*1 receptor in colonic wall of STZ-induced diabetic rats were upregulated. The CRT (high dose) could significantly decrease the expressions of AGE, RAGE, TGF-*β*1, and TGF-*β*1 receptor in the diabetic colonic wall. Furthermore, we found that the expressions of AGE, RAGE, TGF-*β*1, and TGF-*β*1 receptor were associated with most morphological and biomechanical parameters of rat colon. In one previous study, we demonstrated that tangweian jianji (high dose) treatment partly restored the morphometric and biomechanical remodeling of the upper gastrointestinal tract in diabetic rats, and one mechanism was through decreasing the mRNA level of RAGE in the GI wall [59]. Therefore we believed that the corrective effect of CRT (high dose) on the expressions of AGE, RAGE, TGF-*β*, and TGF-*β* receptor is one of the molecular pathways for the improvement of CRT on the colon remodeling in diabetic rats.

Furthermore, we demonstrated that high dose of CRT could significantly decrease the blood glucose level in the diabetic rats [13]. The evidence of correlation analysis showed that the blood glucose level highly associated with the expressions of AGE and RAGE. Therefore, the effect of CRT on the colonic wall remodeling of diabetic rats is likely to be partly from lowing blood glucose level. The colonic wall remodeling in diabetes will alter the relative position of the mechanosensitive afferents (zero setting of the mechanosensitive afferents) and their environment [10]. Improving the colonic wall remodeling induced diabetes will improve colon sensory-motor function and further improve the symptoms of the patients in the clinic.

## 5. Conclusion and Perspectives of the Study

STZ-induced diabetes upregulated the expression of AGE, RAGE, TGF-*β*1, and TGF-*β*1 receptor in different colon layers of rats. CRT could reverse the abnormal expressions of AGE, RAGE, TGF-*β*1, and TGF-*β*1 receptor in the diabetic colon. The expressions of those proteins were highly associated with histomorphometric and biomechanical parameters of colon. The evidence showed that the corrective effect of CRT (high dose) on the expressions of AGE, RAGE, TGF-*β*1, and TGF-*β*1 receptor is one of the molecular pathways for the improvement of CRT on the colon remodeling in diabetic rats. In the future, the detail molecular pathways for the effect of AGE and TGF-*β*1 on colonic remodeling in diabetes and the relation between RAGE and TGF-*β*1 receptor in the diabetic GI complication are needed to explore.

## Figures and Tables

**Table 1:** Parameters of glucose, body weight, and morphometry of colon.

	Con	DM	T1	T2
Body weight (g)	443.69 ± 5.01	231.22 ± 5.05^*∗∗*^	268.33 ± 6.23^*∗∗*^	237.11 ± 3.75^*∗∗*^
Blood glucose (mmol/L)	5.06 ± 0.04	30.23 ± 0.41^*∗∗*^	24.04 ± 0.56^*∗∗*#^	32.21 ± 0.33^*∗∗*^
Wall thickness (mm)	0.98 ± 0.05	1.23 ± 0.04^*∗∗*^	1.13 ± 0.05^*∗*#^	1.11 ± 0.03^*∗*#^
Wall area (mm^2^)	11.04 ± 0.81	16.55 ± 0.98^*∗∗*^	13.95 ± 0.62^*∗*#^	15.51 ± 0.92^*∗∗*^
Wet weight per unit (g/cm)	0.13 ± 0.02	0.15 ± 0.02^*∗*^	0.15 ± 0.02^*∗*^	0.16 ± 0.02^*∗*^

Compared with Con: ^*∗*^ *P* < 0.05, ^*∗∗*^ *P* < 0.01; compared with DM: ^#^ *P* < 0.05.

**Table 2:** Parameters of biomechanical properties of colon.

	Con	DM	T1	T2
Opening angle (degree)	101.45 ± 8.84	223.66 ± 21.93^*∗∗*^	159.17 ± 12.31^*∗*#^	195.341 ± 17.22^*∗*^
Inner residual strain (unitless)	−0.21 ± 0.02	−0.29 ± 0.03^*∗*^	−0.26 ± 0.02^*∗*^	−0.27 ± 0.03^*∗*^
Outer residual strain (unitless)	0.12 ± 0.02	0.22 ± 0.03^*∗∗*^	0.12 ± 0.01^##^	0.26 ± 0.03^*∗∗*^
Circumferential constant *α* (kPa)	1.13 ± 0.16	2.12 ± 0.28^*∗∗*^	1.87 ± 0.3162^*∗*#^	2.61 ± 0.38^*∗∗*^
Longitudinal constant *α* (kPa)	25.48 ± 4.12	52.34 ± 8.73^*∗∗*^	36.13 ± 2.48^*∗*#^	44.76 ± 5.59^*∗∗*^

Compared with Con: ^*∗*^ *P* < 0.05, ^*∗∗*^ *P* < 0.01; compared with DM: ^#^ *P* < 0.05, ^##^ *P* < 0.01.

**Table 3:** Fractions of AGE, RAGE, TGF-*β*1, and TGF-*β*1 receptor expressions.

Proteins	Layers	Con	DM	T1	T2
AGE	Mucosa	0.135 ± 0.027	0.196 ± 0.028^*∗*^	0.085 ± 0.019^##^	0.138 ± 0.029^#^
	Muscle	0.147 ± 0.040	0.474 ± 0.065^*∗∗*^	0.173 ± 0.069^##^	0.452 ± 0.099^*∗∗*^
	Submucosa	0.032 ± 0.011	0.167 ± 0.035^*∗∗*^	0.049 ± 0.012^##^	0.094 ± 0.012^*∗*#^

RAGE	Mucosa	0.054 ± 0.007	0.113 ± 0.040^*∗∗*^	0.052 ± 0.017^##^	0.150 ± 0.041^*∗∗*^
	Muscle	0.189 ± 0.061	0.378 ± 0.093^*∗∗*^	0.162 ± 0.042^##^	0.423 ± 0.087^*∗∗*^
	Submucosa	0.059 ± 0.033	0.263 ± 0.078^*∗∗*^	0.071 ± 0.021^##^	0.226 ± 0.047^*∗*^

TGF-*β*1	Mucosa	0.061 ± 0.014	0.119 ± 0.036^*∗∗*^	0.065 ± 0.012^##^	0.194 ± 0.062^*∗∗*^
	Muscle	0.211 ± 0.028	0.331 ± 0.072^*∗*^	0.191 ± 0.031^#^	0.344 ± 0.102^*∗*^
	Submucosa	0.097 ± 0.019	0.173 ± 0.051^*∗∗*^	0.099 ± 0.022^##^	0.275 ± 0.083^*∗∗*^

TGF-*β*1 receptor	Mucosa	0.116 ± 0.025	0.232 ± 0.066^*∗∗*^	0.141 ± 0.024^#^	0.278 ± 0.035^*∗∗*^
	Muscle	0.188 ± 0.008	0.422 ± 0.079^*∗∗*^	0.227 ± 0.022^##^	0.403 ± 0.044^*∗∗*^
	Submucosa	0.231 ± 0.023	0.379 ± 0.069^*∗*^	0.222 ± 0.017^#^	0.399 ± 0.021^*∗*^

Compared with Con: ^*∗*^ *P* < 0.05, ^*∗∗*^ *P* < 0.01; compared with DM: ^#^ *P* < 0.05, ^##^ *P* < 0.01.

**Table 4:** The relation between expressions of AGE, RAGE TGF-*β*1, and TGF-*β*1 receptor with other parameters.

Proteins	Linear regression equation	*R* values	*P* values
AGE	AGE-M = 0.0651 + (0.00311 *∗* Glu)	0.527	0.010
	AGE-M = −0.0297 + (0.0115 *∗* W-a)	0.553	0.006
	AGE-M = 0.0690 + (0.375 *∗* Res-out)	0.576	0.004
	AGE-M = 0.0472 + (0.000549 *∗* OA)	0.536	0.008
	AGE-Mu = 0.0367 + (0.0134 *∗* Glu)	0.673	<0.001
	AGE-Mu = −0.540 + (0.802 *∗* W-h)	0.479	0.021
	AGE-Mu = −0.304 + (0.0450 *∗* W-a)	0.640	0.001
	AGE-Mu = 0.0765 + (1.494 *∗* Res-out)	0.679	<0.001
	AGE-Mu = −0.0104 + (0.00219 *∗* OA)	0.632	0.001
	AGE-Mu = 0.167 + (0.0910 *∗* C-*α*)	0.430	0.040
	AGE-Sub = 0.00190 + (0.00426 *∗* Glu)	0.636	0.001
	AGE-Sub = 0.223 − (0.000435 *∗* BW)	0.498	0.015
	AGE-Sub = 0.00176 + (168.500 *∗* Wt)	0.462	0.026
	AGE-Sub = −0.267 + (0.332 *∗* W-h)	0.590	0.003
	AGE-Sub = −0.121 + (0.0153 *∗* W-a)	0.648	<0.001
	AGE-Sub = 0.00494 + (0.526 *∗* Res-out)	0.711	<0.001
	AGE-Sub = −0.0129 + (0.000694 *∗* OA)	0.596	0.003
	AGE-Sub = 0.0254 + (0.0376 *∗* C-*α*)	0.529	0.009

RAGE	RAGE-M = 0.0114 + (0.00418 *∗* Glu)	0.544	0.007
	RAGE-M = 0.229 − (0.000415 *∗* BW)	0.435	0.038
	RAGE-M = −0.00701 + (201.874 *∗* Wt)	0.507	0.014
	RAGE-M = −0.231 + (0.310 *∗* W-h)	0.504	0.014
	RAGE-M = −0.111 + (0.0153 *∗* W-a)	0.636	0.001
	RAGE-M = 0.0507 + (0.340 *∗* Res-out)	0.421	0.046
	RAGE-M = 0.00428 + (0.000625 *∗* OA)	0.529	0.009
	RAGE-Mu = 0.0833 + (0.0107 *∗* Glu)	0.565	0.005
	RAGE-Mu = 0.631 − (0.00104 *∗* BW)	0.443	0.034
	RAGE-Mu = −0.0898 + (0.0296 *∗* W-a)	0.501	0.015
	RAGE-Mu = 0.0857 + (0.00148 *∗* OA)	0.510	0.013
	RAGE-Mu = 0.172 + (0.0826 *∗* C-*α*)	0.438	0.037
	RAGE-Mu = 0.184 + (0.00456 *∗* L-*α*)	0.416	0.048
	RAGE-Sub = −0.0181 + (0.00827 *∗* Glu)	0.630	0.001
	RAGE-Sub = 0.431 − (0.000889 *∗* BW)	0.546	0.007
	RAGE-Sub = −0.0568 + (403.042 *∗* Wt)	0.593	0.003
	RAGE-Sub = −0.547 + (0.576 *∗* W-h)	0.549	0.007
	RAGE-Sub = −0.194 + (0.0258 *∗* W-a)	0.627	0.001
	RAGE-Sub = −0.0213 + (0.00117 *∗* OA)	0.582	0.004
	RAGE-Sub = 0.0663 + (0.00336 *∗* L-*α*)	0.441	0.035

TGF-*β*1	TGF-*β*1-M = 0.0129 + (0.00553 *∗* Glu)	0.564	0.009
	TGF-*β*1-M = 0.285 − (0.000501 *∗* BW)	0.431	0.040
	TGF-*β*1-M = 0.0415 + (0.0489 *∗* C-*α*)	0.474	0.022
	TGF-*β*1-M = 0.0622 + (0.00235 *∗* L-*α*)	0.447	0.049

TGF-*β*1 receptor	TGFR-M = 0.0894 + (0.00425 *∗* Glu)	0.443	0.034
	TGFR-M = 0.330 − (0.000495 *∗* BW)	0.435	0.038
	TGFR-M = 0.0294 + (275.036 *∗* Wt)	0.531	0.009
	TGFR-M = −0.197 + (0.346 *∗* W-h)	0.447	0.033
	TGFR-M = −0.114 + (0.0208 *∗* W-a)	0.609	0.002
	TGFR-M = 0.0637 + (0.684 *∗* Res-out)	0.654	<0.001
	TGFR-M = 0.0687 + (0.000705 *∗* OA)	0.431	0.040
	TGFR-Mu = 0.123 + (0.00785 *∗* Glu)	0.627	0.001
	TGFR-Mu = 0.522 − (0.000753 *∗* BW)	0.507	0.013
	TGFR-Mu = 0.113 + (334.580 *∗* Wt)	0.495	0.016
	TGFR-Mu = −0.0774 + (0.0264 *∗* W-a)	0.593	0.003
	TGFR-Mu = 0.188 + (0.645 *∗* Res-out)	0.473	0.023
	TGFR-Mu = 0.104 + (0.00119 *∗* OA)	0.559	0.006
	TGFR-Sub = 0.190 + (0.00488 *∗* Glu)	0.479	0.021
	TGFR-Sub = 0.449 − (0.000506 *∗* BW)	0.419	0.047
	TGFR-Sub = 0.160 + (248.342 *∗* Wt)	0.451	0.031
	TGFR-Sub = 0.0479 + (0.0176 *∗* W-a)	0.486	0.019
	TGFR-Sub = 0.208 + (0.526 *∗* Res-out)	0.473	0.023
	TGFR-Sub = 0.185 + (0.000700 *∗* OA)	0.415	0.049

Notes: M, mucosa; Mu, muscle; Sub, submucosa; Glu, glucose; BW, body weight; Wt, weight per unit length; W-h, wall thickness; W-a, wall area; Res-in, inner residual strain; Res-out, outer residual strain; OA, opening angle, C-*α*, circumferential constant *α*; L-*α*, longitudinal constant *α*.

**Table 5:** Multiple linear regression analysis for the relation between expressions of AGE, RAGE, TGF-*β*1, and TGF-*β*1 receptor with other parameters.

Parameter	Multiple linear regression equation	*R* values	*F* values	*P* values	Independent *P*	
Glucose	Glu = 8.960 + (57.741 *∗* AGE-M) + (82.058 *∗* RAGE-M) + (10.561 *∗* TGF-M) − (26.793 *∗* TGFR-M)	0.696	3.201	0.032	AGE-M RAGE-M TGF-M TGFR-M	0.103 0.042 0.671 0.371
	Glu = 12.108 + (26.395 *∗* AGE-Mu) + (23.614 *∗* RAGE-Mu) − (4.778 *∗* TGF-Mu) − (12.056 *∗* TGFR-Mu)	0.762	4.693	0.007	AGE-Mu RAGE-Mu TGF-Mu TGFR-Mu	0.037 0.086 0.765 0.552
	Glu = 13.082 + (48.264 *∗* AGE-Sub) + (42.375 *∗* RAGE-Sub) + (12.137 *∗* TGF-Sub) − (15.258 *∗* TGFR-Sub)	0.748	4.314	0.010	AGE-Sub RAGE-Sub TGF-Sub TGFR-Sub	0.197 0.030 0.523 0.522

Wall area	W-a = 10.385 + (12.595 *∗* AGE-M) + (29.805 *∗* RAGE-M) + (14.784 *∗* TGF-M) − (8.557 *∗* TGFR-M)	0.797	5.909	0.002	AGE-M RAGE-M TGF-M TGFR-M	0.160 0.006 0.030 0.268
	W-a = 10.615 + (10.314 *∗* AGE-Mu) + (1.876 *∗* RAGE-Mu) + (1.158 *∗* TGF-Mu) + (2.991 *∗* TGFR-Mu)	0.706	3.377	0.027	AGE-Mu RAGE-Mu TGF-Mu TGFR-Mu	0.016 0.668 0.828 0.656
	W-a = 10.180 + (21.961 *∗* AGE-Sub) + (6.459 *∗* RAGE-Sub) + (0.864 *∗* TGF-Sub) + (5.357 *∗* TGFR-Sub)	0.746	4.258	0.011	AGE-Sub RAGE-Sub TGF-Sub TGFR-Sub	0.062 0.253 0.881 0.463

Outer residual strain	res-out = 0.0647 + (0.557 *∗* AGE-M) + (0.706 *∗* RAGE-M) + (0.663 *∗* TGF-M) − (0.463 *∗* TGFR-M)	0.808	6.411	0.002	AGE-M RAGE-M TGF-M TGFR-M	0.039 0.021 0.002 0.048
	res-out = 0.104 + (0.377 *∗* AGE-Mu) − (0.0391 *∗* RAGE-Mu) − (0.0109 *∗* TGF-Mu) − (0.101 *∗* TGFR-Mu)	0.697	3.220	0.032	AGE-Mu RAGE-Mu TGF-Mu TGFR-Mu	0.005 0.766 0.945 0.619
	res-out = 0.0843 + (1.013 *∗* AGE-Sub) − (0.0550 *∗* RAGE-Sub) + (0.167 *∗* TGF-Sub) − (0.0130 *∗* TGFR-Sub)	0.738	4.076	0.013	AGE-Sub RAGE-Sub TGF-Sub TGFR-Sub	0.007 0.742 0.345 0.953

Opening angle	OA = 100.128 + (219.169 *∗* AGE-M) + (463.871 *∗* RAGE-M) + (218.084 *∗* TGF-M) − (101.125 *∗* TGFR-M)	0.643	2.394	0.081	AGE-M RAGE-M TGF-M TGFR-M	0.337 0.078 0.196 0.607
	OA = 81.341 + (200.133 *∗* AGE-Mu) − (27.827 *∗* RAGE-Mu) − (105.944 *∗* TGF-Mu) + (299.716 *∗* TGFR-Mu)	0.814	6.690	0.001	AGE-Mu RAGE-Mu TGF-Mu TGFR-Mu	0.007 0.704 0.243 0.015
	OA = 66.919 + (310.291 *∗* AGE-Sub) + (112.010 *∗* RAGE-Sub) − (109.444 *∗* TGF-Sub) + (276.150 *∗* TGFR-Sub)	0.716	3.576	0.022	AGE-Sub RAGE-Sub TGF-Sub TGFR-Sub	0.204 0.351 0.380 0.088

C-constant *α*	C-*α* = 1.106 + (2.058 *∗* AGE-M) + (3.470 *∗* RAGE-M) + (4.103 *∗* TGF-M) − (0.635 *∗* TGFR-M)	0.499	1.127	0.384	AGE-M RAGE-M TGF-M TGFR-M	0.587 0.415 0.149 0.847
	C-*α* = 0.851 + (2.003 *∗* AGE-Mu) + (1.678 *∗* RAGE-Mu) + (2.208 *∗* TGF-Mu) − (0.0908 *∗* TGFR-Mu)	0.608	1.991	0.132	AGE-Mu RAGE-Mu TGF-Mu TGFR-Mu	0.145 0.266 0.230 0.968
	C-*α* = 0.845 + (6.500 *∗* AGE-Sub) + (0.222 *∗* RAGE-Sub) + (1.132 *∗* TGF-Sub) + (1.359 *∗* TGFR-Sub)	0.607	1.985	0.133	AGE-Sub RAGE-Sub TGF-Sub TGFR-Sub	0.119 0.911 0.588 0.604

L-constant *α*	L-*α* = 12.292 + (151.852 *∗* AGE-M) − (12.161 *∗* RAGE-M) − (19.859 *∗* TGF-M) − (3.768 *∗* TGFR-M)	0.560	1.555	0.226	AGE-M RAGE-M TGF-M TGFR-M	0.028 0.866 0.673 0.946
	L-*α* = 13.874 + (32.678 *∗* AGE-Mu) + (34.214 *∗* RAGE-Mu) + (55.419 *∗* TGF-Mu) − (13.298 *∗* TGFR-Mu)	0.628	2.119	0.113	AGE-Mu RAGE-Mu TGF-Mu TGFR-Mu	0.176 0.200 0.094 0.740
	L-*α* = 21.304 + (4.899 *∗* AGE-Sub) + (57.401 *∗* RAGE-Sub) + (32.987 *∗* TGF-Sub) − (21.109 *∗* TGFR-Sub)	0.526	1.301	0.309	AGE-Sub RAGE-Sub TGF-Sub TGFR-Sub	0.949 0.145 0.411 0.672

Notes: M, mucosa; Mu, muscle; Sub, submucosa; Glu, glucose; W-a, wall area; Res-out, outer residual strain; OA, opening angle, C-*α*, circumferential constant *α*; L-*α*, longitudinal constant *α*.

**Table 6:** Interrelation among AGE, RAGE, TGF-*β*1, and TGF-*β*1 receptor expressions.

Parameter	Multiple linear regression equation	*R* values	*F* values	*P* values	Independent *P*	
Mucosa-AGE	AGE-M = 0.0812 + (0.372 *∗* RAGE-M) + (0.0661 *∗* TGF-M) − (0.0259 *∗* TGFR-M)	0.431	1.026	0.421	RAGE-M TGF-M TGFR-M	0.173 0.660 0.903

Muscle-AGE	AGE-Mu = −0.0919 + (0.640 *∗* RAGE-Mu) + (0.161 *∗* TGF-Mu) + (0.409 *∗* TGFR-Mu)	0.726	5.004	0.007	RAGE-Mu TGF-Mu TGFR-Mu	0.012 0.576 0.329

Submucosa-AGE	AGE-Sub = −0.0559 + (0.294 *∗* RAGE-Sub) + (0.134 *∗* TGF-Sub) + (0.185 *∗* TGFR-Sub)	0.707	4.492	0.011	RAGE-Sub TGF-Sub TGFR-Sub	0.014 0.336 0.272

Mucosa-RAGE	RAGE-M = 0.0390 + (0.271 *∗* AGE-M) − (0.135 *∗* TGF-M) + (0.492 *∗* TGFR-M)	0.785	7.235	0.001	AGE-M TGF-M TGFR-M	0.173 0.284 0.002

Muscle-RAGE	RAGE-Mu = 0.232 + (0.477 *∗* AGE-Mu) − (0.367 *∗* TGF-Mu) + (0.423 *∗* TGFR-Mu)	0.752	5.846	0.003	AGE-Mu TGF-Mu TGFR-Mu	0.012 0.129 0.240

Submucosa-RAGE	RAGE-Sub = 0.302 + (0.985 *∗* AGE-Sub) − (0.363 *∗* TGF-Sub) − (0.00665 *∗* TGFR-Sub)	0.720	4.842	0.008	AGE-Sub TGF-Sub TGFR-Sub	0.014 0.147 0.983

Mucosa-TGF-*β*1	TGF-M = 0.0934 + (0.166 *∗* AGE-M) − (0.469 *∗* RAGE-M) + (0.608 *∗* TGFR-M)	0.501	1.511	0.241	AGE-M RAGE-M TGFR-M	0.660 0.284 0.057

Muscle-TGF-*β*1	TGF-Mu = 0.145 + (0.110 *∗* AGE-Mu) − (0.335 *∗* RAGE-Mu) + (0.809 *∗* TGFR-Mu)	0.664	3.553	0.026	AGE-Mu RAGE-Mu TGFR-Mu	0.576 0.129 0.012

Submucosa-TGF-*β*1	TGF-Sub = 0.212 + (0.384 *∗* AGE-Sub) − (0.311 *∗* RAGE-Sub) + (0.309 *∗* TGFR-Sub)	0.670	3.669	0.0242	AGE-Sub RAGE-Sub TGFR-Sub	0.336 0.147 0.279

Mucosa-TGF-*β*1 receptor	TGFR-M = 0.0521 − (0.0329 *∗* AGE-M) + (0.860 *∗* RAGE-M) + (0.307 *∗* TGF-M)	0.778	6.882	0.002	AGE-M RAGE-M TGF-M	0.903 0.002 0.057

Muscle TGF-*β*1 receptor	TGFR-Mu = 0.145 + (0.129 *∗* AGE-Mu) + (0.179 *∗* RAGE-Mu) + (0.375 *∗* TGF-Mu)	0.785	7.245	0.001	AGE-Mu RAGE-Mu TGF-Mu	0.329 0.240 0.012

Submucosa-TGF-*β*1 receptor	TGFR-Sub = 0.366 + (0.361 *∗* AGE-Sub) − (0.00387 *∗* RAGE-Sub) + (0.210 *∗* TGF-Sub)	0.757	6.055	0.003	AGE-Sub RAGE-Sub TGF-Sub	0.272 0.983 0.279

Notes: M, mucosa; Mu, muscle; Sub, submucosa.
